# Progress on elimination of lymphatic filariasis in Sierra Leone

**DOI:** 10.1186/s13071-018-2915-4

**Published:** 2018-06-04

**Authors:** Joseph B. Koroma, Santigie Sesay, Abdul Conteh, Jusufu Paye, Mohamed Bah, Mustapha Sonnie, Mary H. Hodges, Yaobi Zhang

**Affiliations:** 1 Family Health International 360, Ghana Country Office, Accra, Ghana; 2 National Neglected Tropical Disease Control Programme, Ministry of Health and Sanitation, Freetown, Sierra Leone; 3 Helen Keller International, Freetown, Sierra Leone; 4 Helen Keller International, Regional Office for Africa, Dakar, Senegal

**Keywords:** Lymphatic filariasis, *Wuchereria bancrofti*, Neglected tropical disease, Mass drug administration, Pre-transmission assessment survey, Sierra leone

## Abstract

**Background:**

A baseline survey in 2007–2008 found lymphatic filariasis (LF) to be endemic in Sierra Leone in all 14 districts and co-endemic with onchocerciasis in 12 districts. Mass drug administration (MDA) with ivermectin started in 2006 for onchocerciasis and was modified to add albendazole in 2008 to include LF treatment. In 2011, after three effective MDAs, a significant reduction in microfilaraemia (mf) prevalence and density was reported at the midterm assessment. After five MDAs, in 2013, mf prevalence and density were again measured as part of a pre-transmission assessment survey (pre-TAS) conducted per WHO guidelines.

**Methods:**

For the pre-TAS survey, districts were paired to represent populations of one million for impact assessment. One sentinel site selected from baseline and one spot check site purposefully selected based upon local knowledge of patients with LF were surveyed per pair (two districts). At each site, 300 people over five years of age provided mid-night blood samples and mf prevalence and density were determined using thick blood film microscopy. Results are compared with baseline and midterm data.

**Results:**

At pre-TAS the overall mf prevalence was 0.54% (95% CI: 0.36–0.81%), compared to 0.30% (95% CI: 0.19–0.47) at midterm and 2.6% (95% CI: 2.3–3.0%) at baseline. There was a higher, but non-significant, mf prevalence among males *vs* females. Eight districts (four pairs) had a prevalence of mf < 1% at all sites. Two pairs (four districts) had a prevalence of mf > 1% at one of the two sites: Koinadugu 0.98% (95% CI: 0.34–2.85%) and Bombali 2.67% (95% CI: 1.41–5.00%), and Kailahun 1.56% (95% CI: 0.72–3.36%) and Kenema 0% (95% CI: 0.00–1.21%).

**Conclusions:**

Compared to baseline, there was a significant reduction of LF mf prevalence and density in the 12 districts co-endemic for LF and onchocerciasis after five annual LF MDAs. No statistically significant difference was seen in either measure compared to midterm. Eight of the 12 districts qualified for TAS. The other four districts that failed to qualify for TAS had historically high LF baseline prevalence and density and had regular cross-border movement of populations. These four districts needed to conduct two additional rounds of LF MDA before repeating the pre-TAS. The results showed that Sierra Leone continued to make progress towards the elimination of LF as a public health problem.

## Background

Lymphatic filariasis (LF) is a vector-borne disease caused by one of three filarial parasite species, *Wuchereria bancrofti*, *Brugia malayi* and *Brugia timori* [1], and it is transmitted by mosquitoes, mainly the *Anopheles* mosquitoes in West Africa [2, 3]. LF causes physical and emotional suffering from the disabling and disfiguring lesions (such as hydrocoele, lymphoedema, lymphangitis and elephantiasis) and economic loss due to diminished productivity and incapacitation, and affects mainly poor countries and marginalised people [4–6]. The World Health Organization (WHO) estimates 120 million people globally are affected, with an estimated 40 million having clinically significant manifestations and the disease was identified as the second most common cause of long-term disability [7, 8].

In 1993 the International Task Force on Disease Eradication identified LF as one of six diseases that could be eradicated globally based on available diagnostic tools and strategies. The World Health Assembly passed resolution WHA 50.29 in 1997 calling for LF elimination as a public health problem globally by the year 2020. Subsequently, the WHO launched the Global Programme to Eliminate LF (GPELF) in 2000 to support endemic countries and a Global Alliance for the Elimination of LF (GAELF) was established [9]. The two principal objectives are an interruption of LF transmission and alleviation/prevention of LF-related disability and suffering [9, 10]. According to the WHO recommendation, the main strategy is annual mass drug administration (MDA) of albendazole (400 mg) together with diethylcarbamazine (6 mg/kg) or ivermectin (200 μg/kg) to those known at-risk populations eligible within endemic areas [8–10]. Annual MDA with a minimum treatment coverage of 65% in the total at-risk population for at least five years is required to achieve the objective (microfilaraemia prevalence to below 1%) [8–10]. In 2015, among the 73 known LF endemic countries, 18 no longer required MDA and were conducting post-MDA surveillance [1]. Togo was confirmed as the first African country to eliminate LF as a public health problem in 2017 [11]. Globally, the estimate for people requiring LF MDA has dropped from 1.41 billion in 2011 to 856.4 million in 2016 [1].

In 2005, the Ministry of Health and Sanitation in Sierra Leone conducted nationwide LF mapping with support from WHO and found that all 14 districts were endemic for LF while 12 rural districts (except Western Areas), were co-endemic with onchocerciasis [12, 13]. The existing National Onchocerciasis Control Programme (NOCP) was expanded in 2007 to become the national integrated Neglected Tropical Disease Programme (NTDP) including LF, schistosomiasis and soil-transmitted helminthiasis [12, 14, 15]. An integrated LF/onchocerciasis MDA was piloted in the same year by adding albendazole to the community-directed treatment with ivermectin (CDTI) platform in six districts. This was expanded in 2008 by the NTDP to all 12 co-endemic districts reaching all targeted communities [15]. MDA results reported by the NTDP between 2008 and 2010 indicated good community compliance: epidemiological coverage (i.e. proportion of people ingesting the LF medicines during treatment among the total population of the endemic communities and districts) was above 65%, programme coverage (i.e. proportion of people ingesting the LF medicines during treatment among the eligible people in the endemic communities and districts) was above 80%, and the geographical coverage (i.e. proportion of communities and districts that were actually treated among the total number of endemic communities and districts) was maintained at 100% [15]. In 2011, a midterm impact assessment was conducted after three annual rounds of MDA in these 12 districts, and the results suggested progress was on track to achieve LF elimination objectives in Sierra Leone [15].

In 2013, a pre-transmission assessment survey (pre-TAS) was conducted in the 12 districts that had received at least five effective rounds of LF MDA. This paper presents the pre-TAS survey results, in comparison with the baseline and midterm data and discusses whether the criteria for conducting a transmission assessment survey (TAS) for stopping LF MDA had been met in districts.

## Methods

### Mass drug administration

Integrated annual onchocerciasis/LF MDA with ivermectin plus albendazole was implemented from 2008 to 2013 in all 12 co-endemic districts. MDA was district-wide covering all villages, towns and district headquarter towns. Within villages, community drug distributors (CDDs) were literate members selected by their communities and trained by district health workers to conduct MDA and report adverse events. The CDDs administered between 1 and 4 ivermectin tablets depending on the height of the person using a dose pole while only one tablet of albendazole was administered to each eligible person. District health workers supervised the CDDs with support from district health management teams (DHMT) and national NTDP staff. The CDTI plus albendazole strategy, which was based on volunteer CDDs, could not work in the urban district headquarter towns where people refused to accept medication from volunteers without formal training. Therefore, students in health and nursing institutions were trained to conduct MDA in headquarter towns [16]. MDA was performed once a year between October and December.

Community registers used previously for onchocerciasis MDA were modified to include albendazole and provided to all targeted villages. The register captured all members of each community, including those eligible for MDA and those not eligible. Before each MDA in rural communities (villages), CDDs conducted a pre-MDA census and updated the community register. MDA details were also captured in the registers. Simple tally sheets were used in urban areas by health and nursing students for recording MDA data. Each level had a summary form for ease of reporting: CDDs and health/nursing students to the supervising staff of peripheral health unit (PHU), PHUs to the DHMT, and DHMTs to the NTDP.

### Survey site selection

WHO guidelines were followed for each survey [17, 18]. At baseline (2007–2008), following national mapping of LF using immunochromatographic test (ICT) cards, one site with the highest ICT prevalence in each district was selected as sentinel site (SS) and the baseline data on microfilariae (mf) were collected [12]. As the population size in most districts was below 500,000, two districts were paired to represent a population close to one million depending on geographical proximity and epidemiological characteristics [12, 17, 18]. At midterm, SS and spot check sites (SCS) were selected and surveyed: one SS and one SCS per pair of districts, as described previously [15]. During pre-TAS in the 12 rural districts, the same SSs as for midterm assessment were surveyed in Bo, Bonthe, Kailahun, Koinadugu, Kono and Port Loko, together with different SCSs purposefully selected in Bombali, Kambia, Kenema, Moyamba, Pujehun and Tonkolili in consultation with DHMTs and PHU staff from communities with high numbers of patients with hydrocoeles or lymphedema. The 2 districts in the Western Area did not implement MDA until 2010 so were not eligible for pre-TAS in 2013. In each of the pairs, an SS was selected in one district and an SCS in the other. Since Bombali was the only district with greater than 1% mf prevalence at mid-term assessment after three effective rounds of MDA [15], two SCSs were selected in that district.

### Sampling and diagnosis

In all surveys, convenience sampling was used at each site [12, 15]. Two-day training was conducted for all technicians before the study started to ensure standardisation of activities and data recording. Upon arrival in communities the survey teams first met with community leaders to obtain their approval, then meetings were held with the community to explain the study and its significance. The coordinates of each study site were recorded using hand-held global positioning system units.

A minimum of 300 participants over five years of age were required for pre-TAS [17], so if the sample sizes could not be reached at the primary villages, the survey teams moved to neighbouring villages until the sample sizes were met. Night blood survey methodology through the preparation of thick blood film for microscopy was used [12, 15]. A fingertip blood sample (60 μl) was collected from each participant between 22:00 h and 02:00 h, smeared gently and uniformly in a circular shape onto a slide and allowed to air dry at room temperature for 12–24 h. The next day, the dried smear was dehaemoglobinized through flooding with distilled water for 3–5 min, air-dried again, fixed with methanol for 30–60 s, stained with GIEMSA for 10 min, and examined for microfilariae (mf) under a light microscope by experienced technicians. Positive findings of mf were recorded, and individual density of infection was calculated and expressed as the number of mf per ml of blood. For quality control, all positive slides and 10% of the negative slides were preserved and examined later by an experienced researcher.

### Statistical analysis

Data were recorded in Microsoft Excel and analysed in SPSS (IBM, Version 23). Prevalence and density of mf were calculated for all 12 districts and compared with the midterm and baseline data previously published [12, 15]. The 95% confidence intervals (CI) for prevalence were calculated using the Wilson score method without continuity correction [19]. The arithmetic mean density of infection with 95% CI was calculated for the total population examined and for positives-only. The Chi-square test was used to compare the differences in prevalence, and the Kruskal-Wallis test was used to compare the differences in density. Differences in prevalence and density were considered significant when *P* < 0.05 [12].

The total population used in rural areas was the total number of people recorded in community registers during the pre-MDA census, while the total population used in urban areas was the figure projected from the 2004 national census [20], with an annual growth rate of 2.5%. A point prevalence map showing geographical locations of the survey sites and results was produced with ArcGIS software (ESRI, version 10.4) [12, 21].

## Results

### MDA results 2011–2012

MDA results for 2008–2010 were published previously [15], and are not shown in this paper, while MDA results for 2011–2012 are shown in Table 1. In total, 14,253 villages and urban areas were treated in the 12 districts each year in 2011–2012, which represents 100% geographical coverage for endemic villages and urban areas. Over four million people were targeted annually. Overall epidemiological coverage was 75.9% and 79.6% in 2011 and 2012, respectively, and was over 65% in each district in each round. The overall programme coverage was 94.9% and 93.6% in 2011 and 2012, respectively, and was over 80% in each district in each round. Similar effective MDA coverage was reported for 2008–2010 [15].

**Table 1:** Lymphatic filariasis MDA results in 12 districts of Sierra Leone in 2011 and 2012. Geographical coverage of villages/urban areas was 100% in all 12 districts in 2011 and 2012

District	2011					2012				
	Population			MDA Coverage (%)		Population			MDA Coverage (%)	
	Eligable	Total	Treated	Epidemiological	Programme	Eligable	Total	Treated	Epidemiological	Programme
Bo	444,317	555,397	427,682	77.0	96.3	483,417	568,727	449,508	79.0	93.0
Bombali	390,424	488,030	366,980	75.2	94.0	424,781	499,743	399,794	80.0	94.1
Bonthe	118,597	148,246	112,424	75.8	94.8	128,703	151,416	120,640	79.7	93.7
Kailahun	343,508	429,386	335,567	78.2	97.7	373,737	439,691	349,889	79.6	93.6
Kambia	258,571	323,214	244,376	75.6	94.5	281,326	330,972	263,822	79.7	93.8
Kenema	488,245	610,307	463,162	75.9	94.9	531,550	625,354	501,280	80.2	94.3
Koinadugu	300,392	375,491	282,735	75.3	94.1	326,826	384,502	307,878	80.1	94.2
Kono	358,286	447,858	342,241	76.4	95.5	389,816	458,608	364,975	79.6	93.6
Moyamba	261,017	326,272	238,818	73.2	91.5	283,987	334,103	264,863	79.3	93.3
Port Loko	399,995	499,994	378,976	75.8	94.7	434,034	510,629	403,508	79.0	93.0
Pujehun	188,875	236,094	176,924	74.9	93.7	205,496	241,760	192,140	79.5	93.5
Tonkolili	341,039	426,299	325,639	76.4	95.5	370,702	436,121	345,643	79.3	93.2
Total	3,893,266	4,866,588	3,695,524	75.9	94.9	4,234,375	4,981,626	3,963,940	79.6	93.6

### Microfilaraemia prevalence

At pre-TAS a total of 4230 night blood samples were collected: males 2275 (53.8%), females 1955 (46.2%). The pre-TAS results for each district are shown in Table 2 and compared to baseline and midterm by mf prevalence, arithmetic mean mf density for persons tested positive only (AMD-positives) and arithmetic mean mf density for all persons tested (AMD-all).

**Table 2:** Summary results of LF studies in 12 districts of Sierra Leone at baseline, midterm and pre-TAS

Districts by Evaluation unit		Baseline survey 2007–2008 (95% CI)				Midterm 2011 (95% CI)				Pre-TAS 2013 (95% CI)			
		*n*	mf Prev (%)	AMD-all (mf/ml)	AMD-positive (mf/ml)	*n*	mf Prev (%)	AMD-all (mf/ml)	AMD-positive (mf/ml)	*n*	mf Prev (%)	AMD-all (mf/ml)	AMD-positive (mf/ml)
Overall		8233	2.6 (2.3–3.0)	1.32 (1.00–1.65)	50.90 (40.25–61.62)	6023	0.30 (0.19–0.47)	0.05 (0.03–0.08)	17.59 (15.64–19.55)	4230	0.54 (0.36–0.81)	1.04 (0.30–1.77)	137.12 (88.80–185.44)
By Sex													
Male		3863	3.3 (2.8–3.9)	1.83 (1.21–2.44)	55.08 (39.00–71.15)	3170	0.35 (0.19–0.62)	0.06 (0.03–0.10)	18.18 (14.80–21.56)	2275	0.70 (0.43–1.14)	1.36 (0.08–2.64)	115.56 (60.68–170.43)
Female		4370	2.0 (1.6–2.4)	0.88 (0.59–1.18)	44.76 (32.89–56.64)	2853	0.25 (0.12–0.51)	0.04 (0.01–0.07)	16.67 (-)	1955	0.36 (0.17–0.74)	0.66 (0.08–1.23)	183.33 (70.06–296.60)
By district and sites													
1	Bo	1005	2.0 (1.3–3.1)	1.97 (0.84–3.11)	99.17 (58.32–140.01)	500	0 (0–0.76)	–	–	350	0.29 (0.05–1.60)	0.29 (0.00–0.85)	100 (-)
	Pujehun	624	0 (0–0.6)	–	–	500	0 (0–0.76)	–	–	305	0.33 (0.06–1.83)	0.11 (0.00–0.32)	33.33 (-)
2	Bonthe	504	1.2 (0.6–2.6)	0.83 (0.02–1.63)	69.44 (13.68–125.21)	499	0.20 (0.04–1.13)	0.03 (0–0.10)	16.67 (-)	309	0 (0–1.23)	–	–
	Moyamba	500	1 (0.4–2.3)	0.67 (0–1.36)	66.67 (6.33–127.00)	500	0 (0–0.76)	–	–	330	0 (0.00–1.15)	–	–
3	Kambia	619	2.1 (1.2–3.6)	0.97 (0.23–1.71)	46.15 (17.04–75.27)	500	0.40 (0.11–1.45)	0.07 (0–0.16)	16.67 (-)	300	0 (0–1.26)	–	–
	Port Loko	500	4.4 (2.9–6.6)	3.53 (1.48–5.59)	80.30 (44.49–116.12)	499	0.20 (0.04–1.13)	0.03 (0–0.10)	16.67 (-)	357	0.28 (0.05–1.57)	0.56 (0.00–1.66)	200 (-)
4	Kono	875	2.4 (1.6–3.6)	1.11 (0.37–1.84)	46.03 (20.09–71.97)	499	0 (0–0.76)	0	0	320	0.63 (0.17–2.25)	0.89 (0.00–2.44)	141.67 (0.00–1518.17)
	Tonkolili	500	2.4 (1.4–4.2)	0.63 (0.24–1.03)	26.39 (17.99–34.79)	523	0.19 (0.03–1.08)	0.03 (0–0.10)	16.67 (-)	316	0 (0–1.20)	–	–
5	Bombali 2	–	–	–	–	–	–	–	–	303	0 (0–1.25)	–	–
	Bombali 1	830	6.9 (5.3–8.8)	1.93 (1.28–2.57)	28.07 (21.70–34.44)	506	1.58 (0.80–3.09)	0.26 (0.08–0.45)	16.67 (-)	337	2.67 (1.41–5.00)	8.21 (0.00–16.93)	175.00 (57.95–292.05)
	Koinadugu	636	5.7 (4.1–7.7)	1.99 (0.95–3.04)	35.19 (19.83–50.54)	498	0.80 (0.31–2.05)	0.17 (0–0.34)	20.83 (7.57– 34.09)	305	0.98 (0.34–2.85)	1.15 (0.00–2.51)	116.67 (7.13–226.21)
6	Kailahun	624	2.6 (1.6–4.1)	2.08 (0.00–4.89)	81.25 (0.00–195.58)	499	0.20 (0.04–1.13)	0.03 (0–0.10)	16.67 (-)	385	1.56 (0.72–3.36)	1.69 (0.00–3.46)	108.33 (2.37–214.29)
	Kenema	1016	0.6 (0.3–1.3)	0.34 (0.00–0.70)	58.33 (4.42–112.24)	500	0 (0–0.76)	–	–	313	0 (0–1.21)	–	–

At pre-TAS the mf prevalence was 0.54% (95% CI: 0.36–0.81%), not significantly different from 0.3% at midterm (*χ*^2^ = 3.741, *df* = 1, *P* > 0.05) but significantly lower than 2.6% at the baseline, a decrease of 79.2% (*χ*^2^ = 63.292, *df* = 1, *P* < 0.0001). The mf prevalence in males 0.70% (95% CI: 0.43–1.14%) was almost twice that in females 0.36% (95% CI: 0.17–0.74%), though the difference was not statistically significant (*χ*^2^ = 2.317, *df* = 1, *P* > 0.05). Similarly, the mf prevalence by sex had slightly increased from midterm (males 0.35%, *χ*^2^ = 3.408, *df* = 1, *P* > 0.05; females 0.25%, *χ*^2^ = 0.508, *df* = 1, *P* > 0.05) but decreased significantly from the baseline (males 3.3%, *χ*^2^ = 42.579, *df* = 1, *P* < 0.0001; females 2.0%, *χ*^2^ = 24.165, *df* = 1, *P* < 0.0001).

**Fig. 1:**
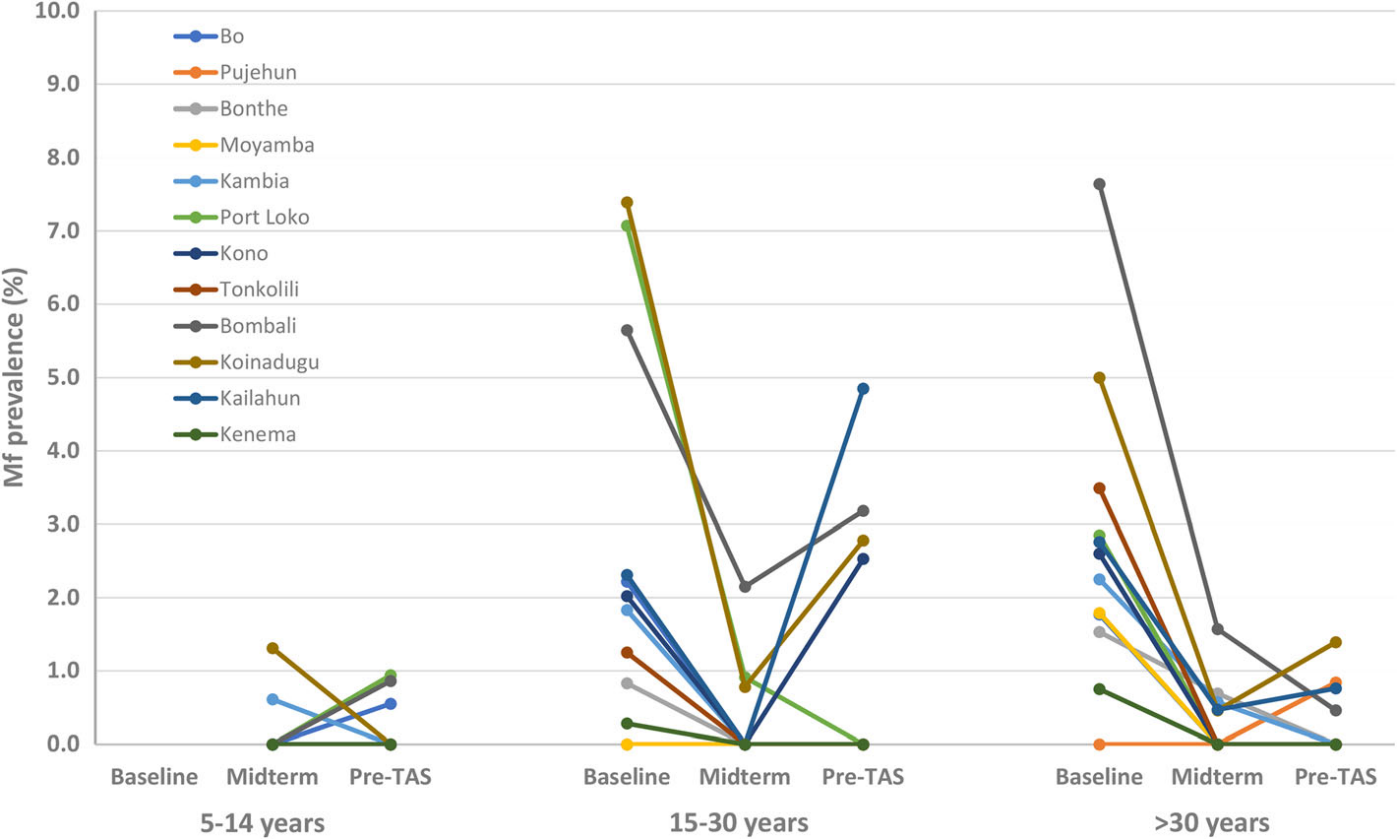
Age mf prevalence curve at baseline, midterm and pre-TAS in each district

The trend of age prevalence in each district at baseline, midterm and pre-TAS is shown in Fig. 1. The people tested at each survey point were divided into three age groups: 5–14 years; 15–30 years; and > 30 years. There were no baseline data for the 5–14 years age group as only people of 15 years and above were tested at baseline. All districts showed major decrease in mf prevalence in two older age groups at midterm from the baseline. At pre-TAS, while most districts showed continuous decrease from midterm in mf prevalence in all age groups, there was a rebound in mf prevalence in certain age groups in a number of districts, most evidently in the 15–30 years group in Bombali, Kailahun, Koinadugu and Kono and in the > 30 years group in Koinadugu which were all > 1%.

### Microfilaraemia density

At pre-TAS the overall AMD-all was 1.04 mf/ml (95% CI: 0.30–1.77 mf/ml) and overall AMD-positive was 137.12 mf/ml (95% CI: 88.80–185.44 mf/ml) as shown in Table 2. For districts, AMD-all was below 1 mf/ml except Bombali, Kailahun and Koinadugu districts. There was no statistically significant difference in mf density in males *vs* females (*H* = 2.308, *df* = 1, *P* > 0.05).

At pre-TAS, the overall AMD-all was not significantly higher than midterm (0.05 mf/ml) (*H* = 3.778, *df* = 1, *P* > 0.05), but significantly lower than the baseline (1.32 mf/ml), a reduction of 21.2% (*H* = 62.810, *df* = 1, *P* < 0.0001). The overall AMD-positive (137.12 mf/ml, 95% CI: 88.80–185.44) was significantly higher than both midterm (17.59 mf/ml) (*H* = 16.625, *df* = 1, *P* < 0.0001) and the baseline (50.90 mf/ml, 95% CI: 40.25–61.62) (*H* = 18.251, *df* = 1, *P* < 0.0001).

### Eligibility of districts for conducting TAS

**Fig. 2:**
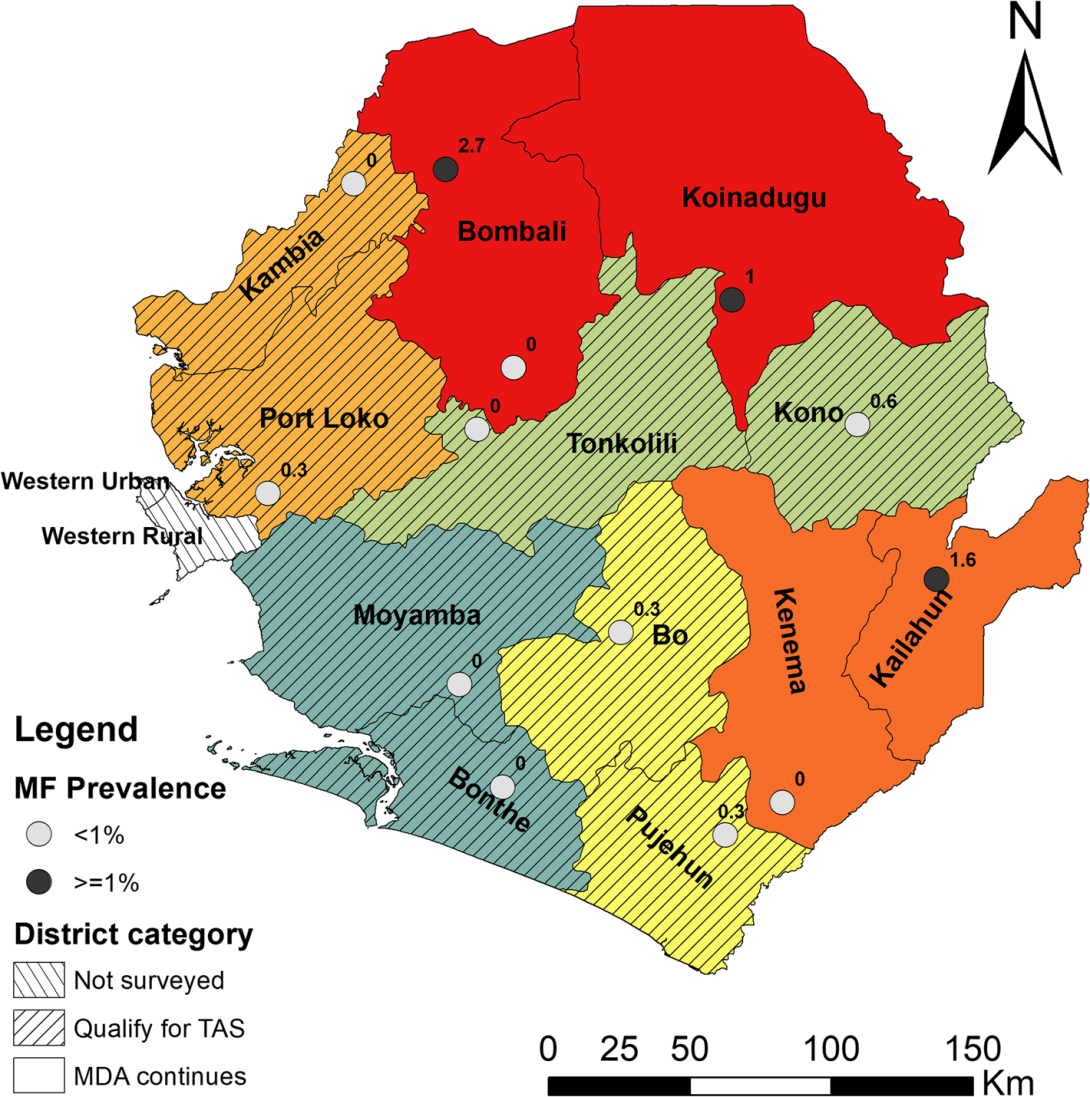
Geographical locations and point mf prevalence of each survey site and district categories for TAS qualification. Paired districts sharing sentinel sites and spot check sites are shown in same colours. Numeral figures at survey sites are point percentage MF prevalence for each site

Prevalence at both SS and SCS were below 1% in Bo-Pujehun (0.3% and 0.3%, respectively), Bonthe-Moyamba (0% and 0%, respectively), Kambia-Port Loko (0% and 0.3%, respectively), and Kono-Tonkolili (0.6% and 0%, respectively) as shown in Fig. 2 and Table 2. These eight districts, therefore, qualified for conducting TAS to confirm whether LF MDA could be stopped. However, in Bombali-Koinadugu districts, the prevalence at three sites was 0%, 2.7% and 1% and in Kailahun-Kenema it was 1.6% and 0% at two sites. These four districts, therefore, failed to meet the criteria for conducting TAS and MDA had to continue for at least two additional rounds.

## Discussion

Our results showed that the criteria for initiating TAS were achieved in eight of twelve districts after five effective rounds of MDA. Mf prevalence declined significantly from 2007–2008 to 2011 and sustained those gains between 2011 and 2013. This indicates that the NTDP continued to make progress towards LF elimination since integrated onchocerciasis/LF MDA using ivermectin/albendazole was piloted in 2007. Many similar studies have been conducted in Kenya, Egypt and Cameroon that have shown a similar significant reduction in LF prevalence and density after five to eight years of LF MDA [22–25]. The results in Sierra Leone were also in line with the expectations of the GPELF [9, 10]. However, four districts still had mf prevalence of over 1% and failed to qualify for conducting TAS.

Prior to MDA, the endemicity of LF in Sierra Leone was one of the highest in Africa [26]. In the early 1990s, surveys showed 34.8% mf prevalence in three villages of Moyamba district [27]. However, in 2007–2008, the pre-MDA mf prevalence for the 12 districts ranged from 0 to 6.9% [12]. This significant reduction may have been partly due to the use of ivermectin for onchocerciasis control, as reported in some other countries [28, 29]. In Sierra Leone, ivermectin was used pre-conflict in limited space in the country [30], but large scale use through CDTI did not start until 1995 as the Special Intervention Zone of the African Programme of Onchocerciasis Control, in meso- and hyper-endemic villages [13]. However, due to the civil conflict between 1991–2002, CDTI did not achieve satisfactory treatment coverage until 2005 and was expanded to accommodate district-wide LF MDA by adding albendazole in 2007–2008 [13]. Therefore, the prior ivermectin use in the 12 dsitricts may have contributed to the reduction of LF prevalence.

It was observed that mf prevalence and overall mf density had dropped significantly at the midterm survey compared to baseline [15] and then increased slightly at pre-TAS. This observation could be due to the convenience sampling strategy that relies on volunteering, and so different sets of the population might have been tested. In addition, the highest mf prevalence was recorded at the purposefully selected SCSs, and particular care had been taken to identify probable hotspots at pre-TAS [31, 32].

Although not statistically significant, almost twice as many males were tested mf positive as females. This may be explained by transmission dynamics as males may be more active and exposed to mosquito bites in the local context, especially since the launch of universal bednet coverage targetting women and children [33]. It has also been suggested that females may be more resistant to LF infection due to hormonal activity [33]. At pre-TAS it was observed that prevalence and density were highest in more active age groups (15–30 years). This may have been due to the continued transmission in those districts that failed to qualify for TAS and mosquito biting rates are higher in these more active age groups [33]. On the other hand, it had been observed that older adolescents and young adults were the most non-compliant to MDA in urban settings in Sierra Leone. It may be that these groups were most concerned about their reproductive health (females unsure about whether or not they were pregnant at the time of MDA) and long-term fertility status, and they may be less aware of the disease-risks than the older age groups who may have seen cases of lymphoedema and hydroceles frequently as they were growing up. The results highlighted the need of improved measures to reach the 15–30 years group in the future MDA in those districts that failed to qualify for conducting TAS.

The number of MDA rounds needed to eliminate LF depends on baseline infection levels, vectoral capacity, the efficacy of the MDA regimen used (ivermectin plus albendazole), and community adherence with MDA [23, 25, 34, 35]. Elimination of LF is achievable in some implementation units with low baseline infection in less than five annual MDAs while more than six annual MDAs may be needed for implementation units with a high baseline prevalence [23, 25, 34, 35]. The marked reduction in prevalence and density in most of the districts after five rounds may have been partly due to the relatively low baseline prevalence [12]. Recent scale-up of insecticidal-treated bed nets (ITNs) and long-lasting insecticidal nets (LLINs) distribution and use, and indoor residual spraying (IRS) for malaria in Sierra Leone may have also benefited the LF results shown in this paper. Over six million ITNs were distributed nationwide in the past five years in Sierra Leone [36–38], and the percentage of households owning mosquito nets increased from 40% in 2008 to 65% in 2013 [38]. IRS was also conducted in selected chiefdoms (sub-district) of four districts: Bo, Bombali, Kono and Rural Western District [38]. The benefit of use of ITNs, LLINs and IRS on LF elimination has been reported in different countries [3, 39–41]. On the other hand, the pre-TAS failure in Bombali and Koinadugu may be explained by the relatively high baseline prevalence and density in the districts. It is also suggested that the pre-TAS failures may have been partly due to cross-border transmission of LF as all four districts that failed are located along the border (Bombali, Koinadugu and Kailahun with Guinea; and Kailahun and Kenema with Liberia) [42–44]. Both Guinea and Liberia had not yet succeeded in reaching 100% geographical coverage for LF MDA and high prevalence rates were recorded in neighbouring Liberia prior to the 1980s [45, 46]. A similar problem of cross-border transmission of LF through migration between Thailand (far advanced with LF elimination) and Myanmar (in its early stages of LF elimination) has been highlighted in several publications [43, 47, 48]. Kailahun had a similar baseline mf prevalence to four other districts that passed the pre-TAS but had remarkably different challenges with cross-border migration with both Guinea and Liberia.

There are several possible limitations of the study. Districts were paired to meet the WHO recommendation of having one SS and one SCS per one million population: one district had an SS while the other had an SCS. This led to fewer sites surveyed per implementation unit (district) as recommended. The results applied to and affected the decision for two districts (implementation units). In the case of Kenema district, although the mf prevalence was below 1% threshold at the site in the district, it could not be considered as having passed the pre-TAS because there was only one site within Kenema and the mf prevalence was above the 1% threshold in the other district of the pair. Furthermore, the districts were paired based on proximity and topographic features, but may not be as similar in relation to transmission dynamics. This district pairing strategy should be reconsidered, and each district should be surveyed separately as an implementation unit in the future. Another limitation is that it was impossible to compare baseline data for the ages 5–14 years because this age group was not studied at baseline per previous WHO guidelines [17, 18].

## Conclusions

There was a significant reduction of LF mf prevalence and density after five annual LF MDAs across the 12 rural districts in Sierra Leone that are co-endemic with onchocerciasis. Eight of 12 districts passed the pre-TAS with < 1% prevalence and qualified for a TAS. The other four districts that failed to qualify for TAS will need to conduct two additional rounds of MDA before repeating the pre-TAS. These promising results for LF were possible because of good community adherence to treatment during MDA campaigns.

## Abbreviations

AFRO: WHO Regional Office for Africa
AMD-all: Arithmetic mean MF density for entire population studied
AMD-positive: Arithmetic mean MF density for positives-only
APOC: African Programme for Onchocerciasis Control
CDD: Community-directed drug distributor
CDTI: Community-directed treatment with ivermectin
CI: Confidence interval
CNTD: Centre for Neglected Tropical Diseases
DHMT: District Health Management Team
GAELF: Global Alliance for the Elimination of LF
GPELF: Global Programme to Eliminate LF
FTS: Filariasis test strip
ICT: Immunochromatographic test
IRS: Indoor residual spraying
ITN: Insecticidal treated nets
LF: Lymphatic filariasis
LLIN: Long-lasting insecticidal nets
MDA: Mass drug administration
MF: Microfilaraemia
mf: Microfilariae
MOHS: Ministry of Health and Sanitation
NBS: Night blood survey
NOCP: National Onchocerciasis Control Programme
NTDP: Neglected Tropical Disease Programme
PHU: Peripheral health unit
Pre-TAS: Pre-transmission assessment survey
SCS: Spot check site
SS: Sentinel site
TAS: Transmission assessment survey
TBF: Thick blood film
USAID: United States Agency for International Development
WHO: World Health Organisation
